# The Role of Work-Family Balance Policy for Enhancing Social Sustainability: A Choice Experiment Analysis of Koreans in their Twenties and Thirties

**DOI:** 10.3390/ijerph16142553

**Published:** 2019-07-17

**Authors:** Inha Oh, Won-Sik Hwang, Hong Jun Yoon

**Affiliations:** 1 Department of Advanced Industry Fusion, Konkuk University, 120 Neungdong-ro Gwangjin-gu, Seoul 143-701, Korea; 2 Korea Institute for Industrial Economics & Trade, 370 Sicheong-daero, Sejong-si 30147, Korea

**Keywords:** population aging, inequality, sustainability, work-family balance, choice experiment, Korea

## Abstract

Korea is facing problems, such as inequality within society and an aging population, that places a burden on public health expenditure. The active adoption of policies that promote work-family balance (WFB), such as parental leave and workplace childcare centers, is known to help solve these problems. However, there has, as yet, been little quantitative evidence accumulated to support this notion. This study used the choice experiment methodology on 373 Koreans in their twenties and thirties, to estimate the level of utility derived from work-family balance policies. The results show that willingness to pay for parental leave was found to be valued at 7.81 million Korean won, while it was 4.83 million won for workplace childcare centers. In particular, WFB policies were found to benefit workers of lower socioeconomic status or belonging to disadvantaged groups, such as women, those with low education levels, and those with low incomes. Furthermore, the utility derived from WFB policies was found to be greater among those who desire children compared to those who do not. The results suggest that the proactive introduction of WFB policies will help solve problems such as inequality within society and population aging.

## 1. Introduction

The Republic of Korea (henceforth “Korea”) is currently facing various challenges in terms of sustainable growth. Among these challenges, the Organization for Economic Co-operation and Development (OECD) [1,2] has pointed to Korea’s rapid pace of population aging and the inequality occurring within its society as major concerns. 

Korean population is currently aging at an exceptionally fast pace. If current trends continue, the old-age support ratio (the population aged 20 to 64 as a ratio to those aged 65 and over) will be around 1.2 by 2062, which is the lowest among OECD economies [3]. This rapid pace of population aging is attributable to Korea’s high average life expectancy and extremely low fertility rate. In particular, Korea currently has the lowest fertility rate among the OECD nations. As of 2018, the total fertility rate is expected to equal only 0.96 [4]. This is a very low figure compared to the average for OECD nations, which was 1.7 as of 2016, and it is among the lowest in the world [5,6]. Aging gives rise to various challenges to a society’s sustainability, such as decreased national tax revenue, reduction in the labor force, and increased expenditures on public health and other infrastructure [7].

The inequality within Korean society manifests itself in various ways. Of these, the OECD’s economic surveys of Korea [1,2] have pointed to several major issues, such as gender inequality, polarization of income levels, and a widening gap between large and Small and Medium sized Enterprises (SMEs) within the economy. Looking at gender inequality, as of 2016, the gap in the median wage between Korean men and women stood at 37%—the largest among the OECD nations and far in excess of the OECD average of 14% [2]. This gender wage gap is attributable to the fact that the burden of childbirth and childcare often falls to women, which leads to career interruption among women. This, in turn, depresses women’s formal participation in the labor market [8]. In addition, polarization in income levels among workers is currently rapidly intensifying in Korea. Workers in the highest 10% income percentile saw their incomes grow by nearly 6% annually from 1997 to 2016, while those in the lowest 10% percentile saw virtually no real wage growth in the same period. As a result, as of 2015, the income level at the highest 10% percentile exceeded that of the lowest 10% percentile by a factor of 4.4, placing Korea’s gap at the 12th-largest among the OECD nations [2]. Polarization between Korea’s major corporations and SMEs is also an ongoing phenomenon. Most SMEs in Korea are subcontractors for major companies, with lower productivity and profitability [9,10]. According to OECD findings [2], as of 2014, the labor productivity of SMEs in the manufacturing sector was only 32.5% that of large firms. This gap, which equaled 53.8% in 1988, has steadily worsened since then. The gap in labor productivity between large firms and SMEs prevents them from offering higher wages. Ultimately, despite Korea’s high youth unemployment rate, 80.5% of Korean SMEs are experiencing difficulty finding employees [2]. According to the OECD [11], such intensification of inequality within society leads to a concentration of wealth and solidification of class stratification, hampering investment in the education and capital of the relatively disadvantaged. In turn, this diminishes creativity and potential, thus undermining the sustainable growth of society as a whole. Also, increased inequality in job quality (which comprises of earnings quality, labor market security and the quality of the work environment) between larger firms and SMEs leads to an occupational health problem and consequently increases the burden on public health expenditure [11]. A report from OECD [11] shows that workers with lower job quality suffers from higher incidence of job strain, as well as higher exposure to physical health risk factors at workplaces. Promoting the quality of job impacts not only on the well-being of workers, but also has direct economic implications, such as higher productivity and lower public health expenditure. 

Meanwhile, the proactive introduction of work-family balance (WFB) policies—including maternity/pregnancy leave, paternity leave, parental leave, working hour reductions for childcare, flexible working hours, and support for the installation of workplace childcare centers—have received attention as measures that could make positive contributions with regard to the many aforementioned sustainable-growth issues currently facing Korea [8,12,13,14,15,16,17].

From the perspective of efforts to boost the fertility rate to address population aging, the lack of a well-established WFB system compels women to choose between their careers and childbirth, ultimately making them averse to have children due to the risk of career interruption associated with childbirth and childcare. Indeed, it is reported that Korean parents have, on average, 0.76 fewer children than they would like to have [18]. Meanwhile, studies have found that sufficient access to a WFB system—such as a guarantee of long-term, paid parental leave and access to reliable public childcare facilities—that supports a balance between one’s career and pregnancy/childbirth is associated with a rise in fertility rates [17]. For instance, during the 1980s, OECD nations saw a decline in their fertility rates as the labor force participation of women rose. However, fertility rates rose again after many countries encouraged both female employment and childbirth by implementing policies to enable WFB. In particular, northern European countries that introduced well-designed and comprehensive WFB policies were able to simultaneously achieve high female employment and fertility rates [17]. A diverse combination of WFB policies has been reported to alleviate the burden of childcare and to prevent the interruption of women’s careers, thereby boosting both the fertility rate and women‘s participation in economic activities and ultimately enhancing gender equality within the labor market [12,13,14,15,16,17]. 

Furthermore, WFB policies are known to provide greater utility to lower-income persons relative to those with higher incomes [17,19]. Those who have high household incomes have the wherewithal to hire alternative caretakers—such as a babysitter—or dedicate themselves full-time to childcare instead of working, thus making them less reliant on publicly-provided WFB policies. On the other hand, members of low-income households have been reported to be more at risk of falling into poverty if childcare entails a reduction in the number of income earners [17]. In such cases, the proactive introduction of WFB policies can help alleviate the negatives effects of income polarization.

Meanwhile, there have been disagreements in the findings of studies regarding the differential benefits provided by WFB policies depending on level of education. Some studies have argued that WFB policies would provide greater utility to persons with higher education levels relative to those with lower education levels, because higher-educated persons would be more career-oriented and thus face higher costs associated with career interruption due to childbirth and childcare [20]. Other studies have argued that higher-educated persons seek prestigious and lucrative positions within their workplace. According to this argument, the use of long vacations—such as parental leave that lasts a few years—would hamper the pursuit of such goals, making higher-educated persons more averse to utilizing WFB policies [21]. Mandel [22] stated that women with low levels of education were likely to be temporarily employed or in physically demanding jobs, thereby having greater need for job security and stable jobs. Thus, Mandel [22] expected that such women would have a strong preference for workplaces with good WFB practices such as paid parental leave programs, which would allow them continued employment, or access to reliable childcare centers. 

As argued above, the active utilization of WFB policies contributes to boosting fertility rates and is expected to help alleviate social inequalities by providing greater benefits to socially disadvantaged groups such as women or low-income earners. For SMEs that have long struggled with recruitment, expanding WFB systems may represent a means of attracting talented workers. Despite the numerous advantages of WFB policies, they do not appear to have been implemented very actively in Korea. According to the “2017 Survey on Work-Family Balance” conducted by the Ministry of Labor [23], only about half of all responding firms (54.5%) reported having implemented parental leave (i.e., up to one year of paid leave for the care of a child aged 8 or under) at least once; this is the most prominent form of WFB policy for the care of infants/young children. Furthermore, there were large differences in implementation depending on firm size—with only about 36% of firms with 30 or fewer employees and approximately 79% of firms with 300 or more employees reporting that they had implemented a parental leave program. Meanwhile, as for workplace childcare centers—which represent an important means of providing reliable childcare after early childhood—implementation rates were even lower. Only about 5% of all firms reported that they were operating a workplace childcare center, either in a proprietary or joint capacity. Differences by firm size were also important in the case of workplace childcare centers—while 18% of firms with 300 or more employees reported that they operated workplace childcare centers, this was true for less than 3% of firms with 100 or fewer employees. The main causes of the low implementation rates of WFB policies are as follows: (1) the cost and difficulty of hiring and retaining replacement workers, (2) a company culture that is unfavorable for WFB policies, and (3) problems with the location and budget for workplace childcare centers [24]. 

The purpose of this study is to quantitatively analyze the extent to which firms practicing WFB policies are preferred by jobseekers relative to firms that do not. From the perspective of firms, the implementation of WFB policies is a costly choice. Therefore, in deciding whether to introduce WFB policies, they need to ascertain, in concrete monetary terms, the extent of the benefits that WFB policies would confer to workers. From the government’s perspective as well, knowledge of workers’ willingness to pay (WTP) for WFB policies would aid in the design of support policies. Furthermore, this study examined how workers’ WTP for WFB policies could differ depending on their various characteristics, such as sex, income level, education level, and pregnancy intention. 

There have been numerous studies on the various ways in which the implementation of WFB policies could affect firms and employees [25,26,27,28]. However, the subjects of these studies have been firms that had already implemented WFB policies and their employees. Therefore, it is difficult to quantitatively determine the extent to which employees prefer WFB policies based on such analyses. Furthermore, mismatches have been observed between jobseekers’ current firms and their workplace preferences, particularly during times of high unemployment [29,30,31]. In view of such limitations, this study employed a choice experiment analysis—a methodology for the analysis of stated preference—to examine how workplace preferences change when firms enact WFB policies. While there have been many studies that have applied choice experiments to analyze jobseekers’ preferences for various workplace attributes [32,33,34,35], few have included the availability of WFB policies among such workplace attributes. This study examines the responses of 373 Korean individuals in their twenties and thirties—age groups that are most active in pursuing job seeking activities while also standing to benefit most from WFB policies—regarding their preferences for hypothetical firms that vary in the extent to which they implement WFB policies.

Among various WFB polices, this study focuses on the availability of parental leave and a workplace childcare center in order to observe the effects of these WFB policies. Although long-term parental leave, which allows workers to return to the workplace after a period of childcare, thus preventing career interruption, is a key component of a WFB system, it is available in only 54.5% of Korean workplaces [23]. In a study conducted by the National Assembly Budget Office [24], which used panel data of 1221 Korean workplaces from 2007 to 2011, regarding which of the various WFB policies contributed to a rise in the ratio of female employees, the availability of parental leave was the only factor to have had a significantly positive effect on this ratio. Thus, in view of its importance, parental leave was included as the principle workplace attribute in terms of WFB policy. The second WFB policy factor was whether there was a workplace childcare center. Because installing a workplace childcare center largely depends on the willingness of the firm to secure the necessary space and funding, and because its availability supports post-infancy childcare, it was included as a factor so that its effect could be examined.

The contributions of this study are as follows. First, it estimates the WTP for WFB policies by using a choice experiment that presented hypothetical firms that varied in their extent of WFB policy implementation. Second, by taking account of jobseeker characteristics (sex, income level, education level, and pregnancy intention), it estimates and compares how groups of jobseekers with different characteristics vary in their perceived WTP for WFB policies. Based on the analyses conducted in this study, we affirm the importance of WFB policy as a valid means of addressing low fertility and inequality, thereby contributing to social sustainability.

## 2. Materials and Methods 

### 2.1. Survey Design

Analyzing preferences for the enactment of WFB policies in the workplace calls for an experiment in which respondents choose from among a few hypothetical firms that combine various workplace attributes with the availability of WFB policies. The analysis in this study employed a discrete choice experiment based on the stated preference of consumers. 

Jobseekers in their twenties and thirties may consider many attributes when choosing a workplace. However, exhaustively accounting for all such attributes is not realistically feasible, nor can rational results be straightforwardly derived from respondents. Therefore, in this study we considered five workplace attributes, as summarized in Table 1. Respondents stated their preferences by choosing from among alternatives constructed by combinations of various levels of the five attributes. In conducting the survey, we assumed that all attributes other than the five specified here remained equal.

Factors such as salary, weekly working hours, and firm size are key workplace attributes that have been considered in previous studies [32,34,35]. The salary attribute was specified in accordance with the usual starting salary at Korean firms, ranging from Korean won (KRW) 20 million to KRW 35 million (1 US dollar = 1141 KRW as of 23 April 2019). As of 2018, the average starting salary among Korean university graduates was KRW 29.5 million, with the salary offered by SMEs being KRW 26.4 million [36]. The intervals of the alternatives were specified to reflect these figures. As per Korea’s Labor Standards Act, the statutory weekly working hours is 40 hours. However, because the number of hours actually worked differs across firms—with some workplaces expecting up to 80 hours of work per week—the levels for this attribute were set at 40 to 80 hours. As of 2012, the average number of hours worked annually by Korean workers was 2163, placing it among the highest ever seen among OECD nations since the early 2000s [8]. Furthermore, as reported by Shin [37], overtime/weekend work is a prevalent phenomenon, with 45% of workers working 50 or more hours per week; 63% of workers are unable to observe the 5-day workweek and 13% work overtime at least three days per week. Because parental leave is strongly encouraged by some firms while being practically unavailable in others, it was defined as a dummy variable. Likewise, the availability of a workplace childcare center was also specified as a dummy variable because some firms operated a childcare center, while others did not. Firm size was defined as a dummy variable (large firm/SME) based on the number of full-time workers and capital. 

From the levels of each attribute in Table 1, 96 combinations are possible (4 × 3 × 2 × 2 × 2 = 96). However, since it would be impossible to analyze respondents’ preferences for all 96 possible alternatives, an orthogonal fractional factorial design that guarantees orthogonality among the levels of each attribute was used to draw 24 combinations. These combinations were then divided into eight choice sets, with three alternatives per set, and the survey was composed to ask respondents to choose their preferred alternative. A sample choice set, as used in the survey, is given in Table 2.

Survey data was collected from Koreans in their 20s and 30s. An offline pilot survey was conducted with 33 respondents on 10 October 2015, followed by an online survey of 340 respondents from April to June of 2016. The online survey was conducted by a professional survey agency. The online survey is conducted using the stratified random sampling where the strata is defined by age, sex and regions (9 provinces and 7 metropolitan cities), and the response rate was 24.2%. We aggregated the pilot survey with the online survey since there has been no substantial changes in economic conditions and policies during the period.

Ultimately, by having 373 (33 + 340) respondents to repeatedly choose from among three hypothetical workplaces eight times, a total of 8952 (373 × 3 × 8) data points were collected. 

It should be noted that the all respondents gave their informed consent for inclusion before they participated in the study. The study was conducted in accordance with the Declaration of Helsinki, and the protocol was approved by the Ethics Committee of Konkuk University (2017-A019-0190).

The demographic characteristics of the respondents are reported in Table 3.

### 2.2. Methodology

We estimated an indirect utility function in order to analyze how key workplace attributes affect preference for workplaces. Using a random utility model, where a respondent’s indirect utility function is determined by workplace attributes and other unobservable characteristics, the probability $Pr_{ij}$ of respondent i choosing workplace j can be written as Equation (1) by applying the logit transformation [38].

(1) $$Pr_{ij}=\frac{\exp\left(V_{ij}\right)}{\sum_{k\in C_{i}}\exp\left(V_{ik}\right)}$$

$V_{ij}$ is the utility of respondent i when he or she chooses workplace j, where the workplace alternatives are composed of combinations of the five attributes, as defined earlier. $C_{i}$ represents the choice set of hypothetical workplaces from which respondent i chooses. In choice experiments, respondents are assumed to make a choice that maximizes their utility given the workplaces presented in the survey. In this study, respondents are presented with three alternatives, whereupon they are made to choose a single most-preferred alternative from among the three based on their workplace attributes. Defining the variable representing the respondent’s choice, $Y_{ij},$ as an indicator function that takes the value of 1 when respondent i chooses workplace j from among the three alternatives given, the log-likelihood function may be written as in Equation (2), given below.

(2) $$\ln L=\sum_{i}^{N}\sum_{j=1}^{3}(Y_{ij}\cdot \ln(Pr_{i}(j|C_{i})),$$

When the observable part of the utility function, $V_{ij}$, is characterized by the attributes of the workplace alternatives and respondent characteristics, the parameters of $V_{ij}$ can be estimated via maximum likelihood methods. The indirect utility function composed of workplace attributes can be expressed as Equation (3).

(3) $$\begin{array}{c} V_{ij}=\beta_{salary}X_{salary\cdot j}+\beta_{working\_hours}X_{working\_hours\cdot j}+\beta_{parental\_leave}X_{parental\_leave\cdot j}+\\ \beta_{childcare\_center}X_{childcare\_center\cdot j}+\beta_{large\_firm}X_{large\_firm\cdot j}, \end{array}$$

Table 4 reports the dummy variables representing the respondent characteristics. Dummy variables have been defined for sex, education level, household income, and pregnancy intention. These variables are specified because these characteristics are expected to affect the extent of utility provided by WFB policies. In terms of sex, respondents were grouped into males (185 persons) and females (188 persons). In terms of education level, respondents were grouped into the high-education group (4-year university or higher: 210 persons) and the low-education group (163 persons). To examine the effects of income levels, respondents were grouped into the high-income group (avg. monthly household income of at least KRW 3 mil.: 237 persons) and the low-income group (136 persons). In terms of pregnancy intention, indicating whether respondents had or planned on having children in the future, respondents were grouped into those who did (*n* = 234 persons) and those who did not (*n* = 139 persons).

If the respondent characteristics are included, the indirect utility function may be written as Equation (4). The example given in Equation (4) shows how the ‘sex’ characteristic of respondents enters the equation. The dummy variables of the other respondent characteristics (education level, household income, and pregnancy intention) enter the equation in the same manner.

(4) $$\begin{array}{c} V_{ij}=\beta_{salary}X_{salary\cdot j}+\beta_{salary\cdot male}X_{salary\cdot j}D_{male\cdot i}+\beta_{working\_hours}X_{working\_hours\cdot j}+\\ \beta_{working\_hours\cdot male}X_{working\_hours\cdot j}D_{male\cdot i}+\beta_{parental\_leave}X_{parental\_leave\cdot j}+\\ \beta_{parental\_leave\cdot male}X_{parental\_leave\cdot j}D_{male\cdot i}+\beta_{childcare\_center}X_{childcare\_center\cdot j}+\\ \beta_{childcare\_center\cdot male}X_{childcare\_center\cdot j}D_{male\cdot i}+\beta_{large\_firm}X_{large\_firm\cdot j}+\\ \beta_{large\_firm\cdot male}X_{large\_firm\cdot j}D_{male\cdot i}, \end{array}$$

Using the estimation results of the indirect utility function, it is possible to estimate each respondent group’s willingness to pay (WTP) for the workplace attributes. For instance, the WTP for weekly working hours can be calculated as in Equation (5), using the parameters in Equation (3). It is possible to calculate the perceived increment in salary associated with changes in weekly working hours by dividing the respondent’s utility increment arising from a unit increase in weekly working hours ($\beta_{working\_hours})$ by the utility increment arising from a unit increase in salary ($\beta_{salary})$. This can be regarded as the respondent’s WTP regarding weekly working hours.

(5) $$\mathrm{WTP}=\frac{\beta_{working\_hours}}{\beta_{salary}},$$

Meanwhile, the parameter estimates from Equation (4) can be used to observe the WTP across respondent characteristics. For instance, the WTP for weekly working hours of males and females, and the difference thereof, can be calculated by Equation (6). The standard errors and statistical significance of the WTP, and the differences thereof, were calculated via bootstrapping (10,000 iterations) [39].

(6) $$\begin{array}{c} \mathrm{WTP}\;\mathrm{of}\;\mathrm{males}=\frac{\beta_{working\_hours}+\beta_{working\_hours\cdot male}}{\beta_{salary}+\beta_{salary\cdot male}}\\ \mathrm{WTP}\;\mathrm{of}\;\mathrm{females}=\frac{\beta_{working\_hours}}{\beta_{salary}}\\ \mathrm{Difference}\;\mathrm{in}\;\mathrm{WTP}=\frac{\beta_{working\_hours}+\beta_{working\_hours\cdot male}}{\beta_{salary}+\beta_{salary\cdot male}}-\frac{\beta_{working\_hours}}{\beta_{salary}}, \end{array}$$

## 3. Results and Discussion

### 3.1. Results

Table 5 reports the estimation results of the indirect utility function in Equation (3). The coefficient estimates for all attributes were significant at the 1% level. The signs of the coefficient estimates were in following with conventional wisdom, with workplaces being more preferred when they offered higher salaries, shorter working hours, were larger firms, and provided access to WFB policies such as parental leave and operational workplace childcare centers. 

The WTP for each workplace attribute is derived as described in Equation (5), the results of which are reported in Table 6. 

The statistical significances of the WTP values were calculated via bootstrapping, with estimates for all attributes being significant at the 1% level. A one-hour decrease in the weekly working hours was associated with a 460,000 won decrease in salary. In hourly terms, this is equivalent to 8846 won—which slightly exceeded the hourly minimum wage (6030 won) at the time of the survey (2016). The availability of parental leave was found to be equivalent to a 7.81 mil. won salary increment relative to a workplace with no parental leave. A workplace with a childcare center was found to be equivalent to a 4.83 mil. won salary increment relative to a workplace with no childcare center. Joining a large firm was found to be equivalent to a 1.03 mil. won salary increment, as opposed to joining an SME. The WTP for firm size was relatively smaller compared to the WTP for WFB policies, suggesting that for SMEs struggling to recruit workers, the adequate provision of WFB policies could serve as an incentive for attracting talented workers. 

Table 7 reports the results of estimating the indirect utility function by gender, so as to examine whether men and women derive different levels of utility from the workplace attributes. The estimation results of the indirect utility function revealed that there were significant differences by gender in the utility derived from weekly working hours and the availability of parental leave. 

In an analogous manner to that given in Equation (6), the results of Table 7 can be used to estimate the WTP by gender for workplace attributes, the results of which are reported in Table 8. 

The standard errors of the WTP values reported in Table 8 were derived via bootstrapping. Looking at the level and difference in men’s and women’s WTP for workplace attributes, women associated a one-hour decrease in weekly working hours with a 584,000 won increase in salary, while the increment was 354,000 won for men. The gender difference in WTP for weekly working hours was approximately 230,000 won, and it was significant at the 1% level. Women derived more utility from parental leave, with a WTP of KRW 10.8 mil., whereas men only have a WTP of KRW 5.24 mil. The difference between genders was large (KRW 5.57 mil.) and highly significant. Meanwhile, the gender differences in WTP for workplace childcare center or firm size were not found to be significant at the 10% level. 

Table 9 reports the estimates of the indirect utility function for the high- and low-education groups, so as to examine whether there were inter-group differences in the utility derived from workplace attributes. 

Using these figures, we calculated each group’s level and difference in terms of WTP for each of the workplace attributes, which are reported in Table 10. 

The most marked difference between the high- and low-education groups appeared to be in the utility derived from the availability of WFB policies. Compared to the-high education group, the low-education group was found to derive higher utility—and thus higher WTP—for WFB policies, with the difference being significant at the 5% level. Among the low-education group, the availability of parental leave was equivalent to an 8.87 mil. won salary increment, and the availability of a workplace childcare center was equivalent to a 6.23 mil. won salary increment. On the other hand, among the high-education group, the WTP for parental leave was estimated to be 7.10 mil. won and that for a workplace childcare center was 3.91 mil. won. 

Using Table 11 and Table 12, we examined whether respondents derived differing levels of utility from workplace attributes depending on their income level. 

The difference owing to income level was most pronounced in the case of the availability of parental leave. Looking at the inter-group difference in WTP, as reported in Table 12, the low-income group had a WTP of KRW 10.95 mil. for the availability of parental leave, while that of the high-income group was KRW 6.28 mil., indicating a highly significant difference of KRW 4.67 mil. 

In Table 13 and Table 14, we examined whether respondents in their 20s and 30s derived differing levels of utility from workplace attributes depending on whether they currently had children or intended to have children in the future (the positive-pregnancy-intention group), relative to those who had no intention of having children in the future (the negative-pregnancy-intention group).

The WTP for WFB policies was found to be higher among those in the positive-pregnancy-intention group. The inter-group WTP differences for parental leave and workplace childcare center were found to be KRW 3.01 mil. and KRW 2.19 mil., respectively. One notable finding was that the negative-pregnancy-intention group preferred joining large companies rather than SMEs, with a WTP difference of KRW 1.39 mil., which was significant at the 10% level.

### 3.2. Discussions

The results of the by-gender analysis adequately reflect the deep-seated perceptions regarding gender roles in Korea [8]. Amid a social atmosphere where achieving WFB is difficult due to long working hours, the burdens of childcare and childbirth are borne by women, who have traditionally performed these roles. As a result, many women have come to prefer workplaces where they can minimize the adversity of career interruption by way of attributes such as shorter working hours or the availability of parental leave, even when these are associated with lower pay. This indicates that proactive implementation of parental leave policy will play an important part in boosting the women’s rate of economic participation in the future.

The results comparing WTPs of groups with different education level show that the low-education group receives larger utility from WFB policies. In Korea, people with lower education levels (high school graduates or those enrolled in/graduated from 2-year colleges) are more likely to take up irregular positions with lower job security [40], where they will be forced to quit in the event of childbirth or childcare constraints. Due to this, they are more averse to having children [41]. Therefore, the low-education group has a much stronger demand for workplaces with higher stability. The estimation results shown here adequately reflect this reality. 

The availability of WFB policies was found to be more important for those with lower incomes. We may infer that people with higher household incomes are less sensitive to the availability of parental leave because they can more easily spare money for alternative means of childcare (e.g., babysitters, etc.) and can also choose to give up work altogether to engage in full-time childcare themselves [24]. Furthermore, lower-income households have been reported to be at risk of poverty when there is a reduction in the number of income earners due to reasons such as childcare [17]. Because of this, lower-income people appear to have a relatively higher preference for workplace attributes that help prevent career interruption, such as the availability of parental leave. 

It has also found that respondents who had children, or intended to have them in the future, were found to have a stronger preference for workplaces with well-established WFB policies. Korea is currently the lowest of all low-fertility countries, with a rate of lower than 1, and 37% of this study’s respondents reported that they had no children and had no plans for childbirth in the future. In line with our expectations, the active implementation of WFB policy provides differentiated support for those with positive pregnancy intentions, implying that it may contribute to reversing the trend of low fertility. Meanwhile, people in their 20s and 30s with no pregnancy intention are more likely to place greater importance on their individual lives and to be more career-oriented, thus preferring larger firms that offer greater opportunities for internal promotion [21,22,42,43].

## 4. Conclusions

While Korea is currently facing various challenges in terms of sustainable development, such as population aging and social polarization, the active implementation of WFB policies at workplaces is expected to be among the measures needed to overcome these challenges. To quantify the extent of benefits that the enactment of WFB policies would provide to the direct beneficiaries—workers in their 20s and 30s—this study conducted choice experiments that gauged the stated preference of 373 respondents regarding hypothetical workplaces. Furthermore, because respondents’ preferences for workplace attributes can vary depending on their individual characteristics, we estimated and compared the WTP for workplace attributes, including WFB policies, for each respondent group (categorized by gender, income level, education level, and pregnancy intention). The key findings of this study are as follows: (1) the WTP for parental leave was found to be as high as KRW 7.81 mil., and WTP for a workplace childcare center was as high as KRW 4.83 mil. Although SMEs were less preferred than large corporations, the implementation of WFB policies could change this preference. (2) Compared to men, women were found to have a stronger preference for workplaces that had shorter working hours and encouraged employees to take parental leave. (3) Relative to the high-education group, those with lower education levels were found to derive significantly greater utility from WFB policies. (4) Relative to the high-income group, the availability of parental leave was found to be more important for those with lower incomes. (5) Respondents who had children, or intended to have them in the future, were found to have a stronger preference for workplaces with well-established WFB policies.

As evident in the above results, participants derive very substantial utility from WFB policies. In particular, WFB policies were found to disproportionately benefit those of lower socioeconomic status or belonging to disadvantaged groups, such as women, the less educated, and those with low incomes. Furthermore, the utility derived from WFB policies was found to be greater among those with positive pregnancy intentions compared to those with negative pregnancy intentions. According to the findings of this study, WFB practices may be regarded as policies with the potential to counteract the major issues facing Korean society—namely, low fertility, population aging, and the intensification of social polarization and inequality. To ensure sustainable development, the Korean government should take a more active stance on implementing WFB policies. From the perspective of firms as well, active adoption of WFB policies can function as an additional incentive, beyond wages, to attract talented workers (women in particular). When considering plans for the active introduction of WFB policies, the actual costs needed to implement the policies should be compared against the WTP estimated in this study.

A relatively small sample size can be a limitation of this study, making it difficult to generalize the results to Korean society. Additionally, according to Orme [44], a typical choice experiment study for business application recommends a sample size of at least 200 for each group when calculating the differences between participant groups. However, in the present study, the number of respondents in subgroup was often less than 200, since the number of entire respondents was only 373. A future study with a larger sample size will enable generalization of the results to the Korean society. 

## Figures and Tables

**Table 1:** Workplace attributes and labels used in the choice experiment.

Attribute	Label	Definition	Attribute Level
Salary	salary	Annual salary before tax.	20 million Korean won (KRW); 25 million KRW; 30 million KRW; 35 million KRW
Weekly working hours	working_hours	Working hours per week.	40 h/week; 60 h/week; 80 h/week
Parental leave	parental_leave	A scheme for which a worker can apply for and utilize to raise a child aged under nine for a period of up to one year. The company must pay 40% of his/her ordinary wage every month during this period.	Unusable (0); Strongly encouraged to utilize (1)
Workplace childcare center	childcare_center	Childcare center inside the facility or jointly operated with nearby companies; more than 50% of the childcare costs are subsidized.	None (0); In operation (1)
Firm size	large_firm	A company is defined as an SME if it has capital of less than 8 billion KRW or fewer than 300 full-time workers. Otherwise, it is a large company.	SME (0); Large company (1)

Note: 1 US dollar = 1141 Korean won (KRW) as of 23 April 2019.

**Table 2:** A sample choice set.

Attribute	Workplace A	Workplace B	Workplace C
Salary	KRW 35 mil.	KRW 35 mil.	KRW 25 mil.
Weekly working hours	40 h	80 h	40 h
Parental leave	Unavailable	Strongly encouraged	Unavailable
Workplace childcare center	Available	Unavailable	Available
Firm size	SME	SME	Large company
Choose the one which you most prefer	( )	( )	( )

**Table 3:** Demographic characteristics of respondents.

Variable	Value	Freq.	Percent (%)
Sex	Female	188	50.4
	Male	185	49.6
Age group	20s	224	60.1
	30s	149	40.0
Total monthly household income (before taxes)	KRW lower than 1 mil.	8	2.1
	KRW 1 mil. or higher ~ under KRW 2 mil.	53	14.2
	KRW 2 mil. or higher ~ under KRW 3 mil.	75	20.1
	KRW 3 mil. or higher ~ under KRW 4 mil.	72	19.3
	KRW 4 mil. or higher ~ under KRW 5 mil.	58	15.6
	KRW 5 mil. or higher ~ under KRW 7 mil.	62	16.6
	KRW 7 mil. or higher	45	12.1
Education level	High school graduate or lower	113	30.3
	Enrolled/graduated 2-year college	50	13.4
	Enrolled/graduated 4-year university	177	47.5
	Graduate school or higher	33	8.9
Pregnancy intention	No intention to have a child in the future.	139	37.3
	Currently has a child (children) or intends to have a child (children) in the future.	234	62.7
Total		373	100.00%

**Table 4:** Dummy variables indicating the respondent characteristics.

Variable	Description
$D_{male\cdot i}$	Dummy variable indicating respondent’s sex Male (=1), Female (=0)
$D_{educ\cdot i}$	Dummy variable indicating respondent’s education level Enrolled/graduated from 4-year university or enrolled or graduated from graduate school (=1), graduated from high school or enrolled/graduated from 2-year college (=0)
$D_{rich\cdot i}$	Household income of respondent Pre-tax monthly income of KRW 3 mil. or higher (=1), Pre-tax monthly income of less than KRW 3 mil. (=0)
$D_{kids\cdot i}$	Currently has a child (children) or intends to have a child (children) in the future (=1), otherwise (=0)

**Table 5:** Estimation results of the indirect utility function.

Variables	Coefficient	S.E.	*p*-Value
$X_{salary}$	0.1401	0.0061	<0.001
$X_{working\_hours}$	−0.0644	0.0024	<0.001
$X_{parental\_leave}$	1.0943	0.0625	<0.001
$X_{childcare\_center}$	0.6759	0.0529	<0.001
$X_{large\_firm}$	0.1446	0.0509	0.0045
AdjR2	0.2805		
Log Likelihood	−2292.3270		

**Table 6:** WTP for workplace attributes.

Attributes	WTP (unit: million KRW)	S.E.	*p*-Value
Weekly working hours	−0.4599	0.0215	<0.001
Parental leave	7.8119	0.4207	<0.001
Workplace childcare center	4.8254	0.4493	<0.001
Firm size	1.0320	0.3687	0.0051

**Table 7:** Estimation results of the indirect utility function, by gender.

Variables	Coefficient	S.E.	*p*-Value
$X_{salary}$	0.1344	0.0090	<0.001
$X_{working\_hours}$	−0.0784	0.0038	<0.001
$X_{parental\_leave}$	1.4533	0.0951	<0.001
$X_{childcare\_center}$	0.6937	0.0760	<0.001
$X_{large\_firm}$	0.0799	0.0718	0.2660
$X_{salary}\times D_{male}$	0.0140	0.0123	0.2554
$X_{working\_hours}\times D_{male}$	0.0259	0.0050	<0.001
$X_{parental\_leave}\times D_{male}$	−0.6758	0.1273	<0.001
$X_{childcare\_center}\times D_{male}$	−0.0156	0.1068	0.8837
$X_{large\_firm}\times D_{male}$	0.1383	0.1028	0.1787
AdjR2	0.2901		
Log Likelihood	−2259.7670		

**Table 8:** WTP for workplace attributes, by gender.

	WTP of Women			WTP of Men			WTP Difference (Men-Women)		
Attributes	WTP (unit: million KRW)	S.E.	*p*-Value	WTP (unit: million KRW)	S.E.	*p*-Value	WTP (unit: million KRW)	S.E.	*p*-Value
Weekly working hours	−0.5836	0.0383	<0.001	−0.3538	0.0248	<0.001	0.2298	0.0456	<0.001
Parental leave	10.8110	0.7201	<0.001	5.2384	0.5261	<0.001	−5.5727	0.8918	<0.001
Workplace childcare center	5.1606	0.6940	<0.001	4.5689	0.5828	<0.001	−0.5916	0.9063	0.5139
Firm size	0.5941	0.5380	0.2695	1.4698	0.5056	0.0036	0.8757	0.7383	0.2356

**Table 9:** Estimation results of the indirect utility function, by level of education.

Variables	Coefficient	S.E.	*p*-Value
$X_{salary}$	0.1375	0.0093	<0.001
$X_{working\_hours}$	−0.0650	0.0038	<0.001
$X_{parental\_leave}$	1.2191	0.0979	<0.001
$X_{childcare\_center}$	0.8570	0.0839	<0.001
$X_{large\_firm}$	0.1161	0.0802	0.1475
$X_{salary}\times D_{educ}$	0.0045	0.0123	0.7145
$X_{working\_hours}\times D_{educ}$	0.0009	0.0049	0.8495
$X_{parental\_leave}\times D_{educ}$	−0.2115	0.1274	0.0969
$X_{childcare\_center}\times D_{educ}$	−0.3013	0.1084	0.0054
$X_{large\_firm}\times D_{educ}$	0.0518	0.1039	0.6183
AdjR2	0.2822		
Log Likelihood	−2284.8180		

**Table 10:** WTP for workplace attributes, by level of education.

	WTP of Low-Education Group			WTP of High-Education Group			WTP Difference (High-Education Group–Low-Education Group)		
Attributes	WTP (unit: million KRW)	S.E.	*p*-Value	WTP (unit: million KRW)	S.E.	*p*-Value	WTP (unit: million KRW)	S.E.	*p*-Value
Weekly working hours	−0.4728	0.0354	<0.001	−0.4512	0.0272	<0.001	0.0216	0.0446	0.6278
Parental leave	8.8686	0.6876	<0.001	7.0969	0.5346	<0.001	−1.7717	0.8709	0.0419
Workplace childcare center	6.2346	0.7805	<0.001	3.9144	0.5485	<0.001	−2.3202	0.9540	0.0150
Firm size	0.8449	0.5905	0.1525	1.1828	0.4726	0.0123	0.3379	0.7563	0.6550

**Table 11:** Estimation results of the indirect utility function, by income level.

Variables	Coefficient	S.E.	*p*-Value
$X_{salary}$	0.1278	0.0101	<0.001
$X_{working\_hours}$	−0.0629	0.0039	<0.001
$X_{parental\_leave}$	1.3993	0.1066	<0.001
$X_{childcare\_center}$	0.6762	0.0882	<0.001
$X_{large\_firm}$	0.1199	0.0841	0.1542
$X_{salary}\times D_{rich}$	0.0201	0.0127	0.1133
$X_{working\_hours}\times D_{rich}$	−0.0032	0.0050	0.5183
$X_{parental\_leave}\times D_{rich}$	−0.4708	0.1320	0.0004
$X_{childcare\_center}\times D_{rich}$	0.0144	0.1105	0.8960
$X_{large\_firm}\times D_{rich}$	0.0546	0.1059	0.6065
AdjR2	0.2846		
Log Likelihood	−2277.3730		

**Table 12:** WTP for workplace attributes, by income level.

	WTP of Low-Income Group			WTP of High-Income Group			WTP Difference (High-Income Group – Low-Income Group)		
Attributes	WTP (unit: million KRW)	S.E.	*p*-Value	WTP (unit: million KRW)	S.E.	*p*-Value	WTP (unit: million KRW)	S.E.	*p*-Value
Weekly working hours	−0.4920	0.0415	<0.001	−0.4471	0.0251	<0.001	0.0449	0.0485	0.3543
Parental leave	10.9464	0.8813	<0.001	6.2761	0.4814	<0.001	−4.6703	1.0042	<0.001
Workplace childcare center	5.2895	0.8521	<0.001	4.6680	0.5259	<0.001	−0.6214	1.0013	0.5349
Firm size	0.9378	0.6682	0.1605	1.1792	0.4412	0.0075	0.2414	0.8007	0.7631

**Table 13:** Estimation results of the indirect utility function, by pregnancy intention.

Variables	Coefficient	S.E.	*p*-Value
$X_{salary}$	0.1524	0.0106	<0.001
$X_{working\_hours}$	−0.0744	0.0042	<0.001
$X_{parental\_leave}$	0.9043	0.1036	<0.001
$X_{childcare\_center}$	0.5402	0.0862	<0.001
$X_{large\_firm}$	0.2836	0.0833	0.0007
$X_{salary}\times D_{kids}$	−0.0167	0.0130	0.1996
$X_{working\_hours}\times D_{kids}$	0.0152	0.0052	0.0033
$X_{parental\_leave}\times D_{kids}$	0.3097	0.1303	0.0175
$X_{childcare\_center}\times D_{kids}$	0.2376	0.1096	0.0301
$X_{large\_firm}\times D_{kids}$	−0.2201	0.1057	0.0373
AdjR2	0.2865		
Log Likelihood	−2271.3280		

**Table 14:** WTP for workplace attributes, by pregnancy intention.

	WTP of Negative-Pregnancy-Intention Group			WTP of Positive-Pregnancy-Intention Group			WTP Difference (Positive-Pregnancy-Intention Group – Negative-Pregnancy-Intention Group)		
Attributes	WTP (unit: million KRW)	S.E.	*p*-Value	WTP (unit: million KRW)	S.E.	*p*-Value	WTP (unit: million KRW)	S.E.	*p*-Value
Weekly working hours	−0.4881	0.0342	<0.001	−0.4358	0.0273	<0.001	0.0522	0.0437	0.2322
Parental leave	5.9322	0.6184	<0.001	8.9424	0.5674	<0.001	3.0102	0.8393	0.0003
Workplace childcare center	3.5441	0.6331	<0.001	5.7301	0.6176	<0.001	2.1860	0.8844	0.0135
Firm size	1.8607	0.5581	0.0009	0.4681	0.4815	0.3309	−1.3927	0.7371	0.0588
